# Spatially varying density dependence drives a shifting mosaic of survival in a recovering apex predator (*Canis lupus*)

**DOI:** 10.1002/ece3.3463

**Published:** 2017-10-28

**Authors:** Shawn T. O'Neil, Joseph K. Bump, Dean E. Beyer

**Affiliations:** ^1^ School of Forest Resources and Environmental Science Michigan Technological University Houghton MI USA; ^2^ Department of Fisheries, Wildlife and Conservation Biology University of Minnesota St. Paul MN USA; ^3^ Wildlife Division Michigan Department of Natural Resources Marquette MI USA; ^4^ Present address: U.S. Geological Survey Western Ecological Research Center 800 Business Park Dr. Suite D Dixon CA, 95620

**Keywords:** landscape of risk, management of endangered species, population recovery, proportional hazards, spatial modeling, species recolonization, survival analysis, Upper Great Lakes wolves, Upper Peninsula

## Abstract

Understanding landscape patterns in mortality risk is crucial for promoting recovery of threatened and endangered species. Humans affect mortality risk in large carnivores such as wolves (*Canis lupus*), but spatiotemporally varying density dependence can significantly influence the landscape of survival. This potentially occurs when density varies spatially and risk is unevenly distributed. We quantified spatiotemporal sources of variation in survival rates of gray wolves (*C. lupus*) during a 21‐year period of population recovery in the Upper Peninsula of Michigan, USA. We focused on mapping risk across time using Cox Proportional Hazards (CPH) models with time‐dependent covariates, thus exploring a shifting mosaic of survival. Extended CPH models and time‐dependent covariates revealed influences of seasonality, density dependence and experience, as well as individual‐level factors and landscape predictors of risk. We used results to predict the shifting landscape of risk at the beginning, middle, and end of the wolf recovery time series. Survival rates varied spatially and declined over time. Long‐term change was density‐dependent, with landscape predictors such as agricultural land cover and edge densities contributing negatively to survival. Survival also varied seasonally and depended on individual experience, sex, and resident versus transient status. The shifting landscape of survival suggested that increasing density contributed to greater potential for human conflict and wolf mortality risk. Long‐term spatial variation in key population vital rates is largely unquantified in many threatened, endangered, and recovering species. Variation in risk may indicate potential for source‐sink population dynamics, especially where individuals preemptively occupy suitable territories, which forces new individuals into riskier habitat types as density increases. We encourage managers to explore relationships between adult survival and localized changes in population density. Density‐dependent risk maps can identify increasing conflict areas or potential habitat sinks which may persist due to high recruitment in adjacent habitats.

## 1 INTRODUCTION

Accurate estimates of key vital rates are crucial for promoting restoration and recovery of threatened and endangered species, especially where humans contribute to changes in population demographics. Anthropogenic impacts have driven many species to the brink of extinction (Vié, Hilton‐Taylor, & Stuart, 2009); however, changes in conservation policy can in some cases allow for recovery. For example, changing perceptions and increased protections have contributed to increases in large carnivore populations over the past several decades (Chapron et al., 2014; Ripple et al., 2014; Smith, Nielsen, & Hellgren, 2016). Gray wolves (*Canis lupus*; Figure S1) are arguably one of the most iconic examples of successful conservation policy (Beschta & Ripple, 2009; Mech & Boitani, 2010; Wydeven et al., 2009). Nonetheless, some wolf population segments remain endangered, in part due to the potential for human actions to reverse positive growth rates or inhibit continued range expansion (Bruskotter, Vucetich, Enzler, Treves, & Nelson, 2014; Liberg et al., 2012; Olson et al., 2015). Similar challenges face other carnivore populations. For example, cougars (*Puma concolor*) have expanded their distribution in the United States recently but have yet to colonize large portions of their historical range due to anthropogenic barriers that limit female dispersal and survival (LaRue & Nielsen, 2008, 2016).

Survival is an important driver of large carnivore populations, especially when humans contribute substantially to mortality. Large carnivores in the United States are frequently subject to legal (e.g., hunting, lethal control), illegal (poaching), and incidental killing (e.g., vehicle strike) (Murray et al., 2010; Stenglein, Van Deelen, et al., 2015; Thompson, Jenks, & Fecske, 2014; Vickers et al., 2015). The relative influence of human‐caused mortality on population dynamics is debated (Creel & Rotella, 2010; Robinson et al., 2014; Stoner, Wolfe, & Choate, 2006) with some subpopulations apparently sustaining high‐mortality rates (Adams, Stephenson, Dale, Ahgook, & Demma, 2008; Creel & Rotella, 2010; Stoner et al., 2006). Monitoring and precise estimation of adult survival in the presence of human‐caused mortality are critical for effective management of large carnivore populations.

Whereas survival estimation on its own is useful and informative for management, understanding explanatory mechanisms is necessary to guide decision making. Annual survival in large carnivores is frequently related to the riskiness of the environment. Greater mortality risk is often associated with the potential for human impacts. For example, wolves inhabiting areas with greater road densities, greater proportions of agricultural land cover, and more private land have lower survival rates than those that occupy remote protected areas (Fuller, Mech, & Cochrane, 2003; Smith et al., 2010; Stenglein, 2014). Similar mosaics of survival occur in cougars and grizzly bears (Johnson, Boyce, Schwartz, & Haroldson, 2004; Ruth et al., 2011; Schwartz, Haroldson, & White, 2010). Survival can also be density‐dependent, especially when populations saturate suitable habitat. In such cases, survival may be regulated by intraspecific aggression and reduced prey availability as groups and individuals compete for territory and prey (Cubaynes et al., 2014; Marucco, Vucetich, Peterson, Adams, & Vucetich, 2012). Characterizing habitat quality in terms of survival is an ecologically informative and valuable tool for resource managers (Gaillard et al., 2010; Mosser, Fryxell, Eberly, & Packer, 2009). Specifically, quantifying spatial variation in survival establishes a critical link between habitat components and individual fitness (DeCesare et al., 2014), providing important information that extends beyond what is typically inferred from traditional habitat selection studies (Matthiopoulos et al., 2015).

Habitat quality can be defined in terms of an environment's potential to provide resources necessary for growth and reproduction and to limit mortality from predation (Matthiopoulos et al., 2015; McLoughlin, Morris, Fortin, Vander Wal, & Contasti, 2010; Morris, 1989; Van Horne, 1983). The combination of environmental characteristics that collectively contributes to net positive population growth constitutes the “true” ecological niche (Pulliam, 2000) which can be difficult to measure directly (Hirzel & Le Lay, 2008; Panzacchi, Van Moorter, Strand, Loe, & Reimers, 2015). A habitat's fitness potential is dynamic and often cannot be inferred from habitat selection or species occurrence studies alone due to density‐dependent habitat‐fitness relationships (McLoughlin, Boyce, Coulson, & Clutton‐Brock, 2006; Morris, 1988) and/or difficulties measuring the true as opposed to the apparent ecological niche (Pulliam, 2000; Van Horne, 1983). Survival modeling with environmental covariates can be a solution to this problem, especially when top‐down influences such as predation risk have strong potential to limit population growth, which is often the case for generalist predators. Relating survival to environmental characteristics can identify habitat‐limiting factors and potential sink habitats that might otherwise go unnoticed, because animals often utilize risky habitats (Aldridge & Boyce, 2007; Battin, 2004; DeCesare et al., 2014). However, estimation of survival rarely accounts for spatiotemporal variation associated with density‐dependent population increase over time. In particular, spatial variation in density is rarely quantified alongside habitat characteristics with similar spatial scales. Density‐dependent interactions with mortality risk factors are likely because fluctuation in density contributes to variation in habitat selection (Matthiopoulos et al., 2015; McLoughlin et al., 2010).

We examined the influence that spatially varying density dependence can have on landscape models of risk, where shifts can occur due to important interactions between suitable habitats that produce high local population densities and adjacent risky habitats that may act as sinks. Using gray wolves as a case study, we evaluated spatiotemporal variation in wolf survival rates in Michigan, USA, from 1992 to 2013. We were interested in obtaining a reliable estimate for adult survival of the population, testing for density‐dependent and/or temporal variation in survival, and evaluating the relative influences on survival within the study area. Hypothesized influences included individual‐level factors (age, sex, body condition at capture, capture type, vaccination status, and pack membership status) and continuous spatial covariates (distance from pack territory, landscape characteristics, spatiotemporally varying density, and movement information). Testing for such effects contributes to (1) evaluation of the factors that increase mortality risk, (2) understanding of how management may influence the population, and (3) knowledge about habitat fitness and potential sink habitats used by wolves. Our approach uses broadly applicable habitat modeling tools that can be extended to any population that is tracked across time.

## 2 METHODS

### 2.1 Field methods

Our study system was the Upper Peninsula (UP) of Michigan, USA. The UP is characterized by dense northern hardwood forests and long winters with heavy snowfall (300–500 cm within lake effect snow belts; Eichenlaub et al., 1990). Human population density and major road densities were generally low throughout the study area (O'Neil, Rahn, & Bump, 2014). Major land uses included mining and logging, with networks of logging roads typically providing recreational access to densely forested land cover types. White‐tailed deer (*Odocoileus virginianus*) were the primary prey source for wolves and were an important seasonally‐varying prey source for other predators (e.g., coyotes [*Canis latrans*], black bear [*Ursus americanus*], and bobcat [*Lynx rufus*]). We refer readers to Beyer, Peterson, Vucetich, and Hammill (2009), O'Neil and Bump (2014), and Potvin et al. (2005) for additional details about the study system.

Wolves were captured using foot‐hold traps during spring and summer, 1992–2013. Capturing efforts were part of an ongoing wolf monitoring and radio‐telemetry program led by the Michigan Department of Natural Resources ([MDNR]; Beyer et al., 2009; MDNR, 2008, 2015). Captures also occurred opportunistically in the fall when wolves were incidentally caught by coyote (*C. latrans*) trappers. Individuals were chemically immobilized (ketamine hydrochloride and xylazine, 100 mg/ml) using 0.11 mg/kg ketamine hydrochloride and 2 mg/kg xylazine and were subsequently sexed, weighed, aged, and fitted with VHF radiocollars (Telonics, Inc., Mesa, AZ, USA; Beyer et al., 2009; Potvin et al., 2005; Vucetich et al., 2012). A few wolves included in the study (*n = *14) were also monitored via GPS collars. We located all collared wolves by fixed‐wing single‐engine aircraft 1–2 times per week during the study. Field crews attempted to physically locate collars shortly after a mortality signal (<1 week). Fate was initially determined via field necropsy, and cause of death was updated as needed via laboratory necropsies at the MDNR Wildlife Disease Laboratory (Lansing, MI, USA).

### 2.2 Wolf packs, territories, and density

The MDNR tracked wolves in winter to complete an annual census of the wolf population and estimate spatial variation in wolf density. All passable roads were surveyed from trucks and snowmobiles (Potvin et al., 2005), beginning in 1992 and continuing through the duration of the study. Once tracks were detected, trackers recorded all signs (territory markings, scat, individual sets of tracks) to estimate pack sizes and establish pack boundaries. The ground tracking abundance estimates were evaluated during a separate 4‐year study (Vucetich et al., 2012), which revealed a 4% average difference between the independent counts. In 2007, the MDNR adopted a geographically stratified sampling plan to reduce cost and effort of the survey, which relied on a panel design to increase precision of abundance estimates and ensure that some of the sampled units were counted during successive years (Beyer et al., 2009; Potvin et al., 2005; Schreuder, 1993). A detailed description of the survey is provided in the Appendix S1.

We used a combination of radio‐collar locations and track survey data to generate annual estimates of wolf density. Once detected, pack territories were monitored either by aerial telemetry relocations from ≥1 resident wolves or by repeatedly visiting known territory sites each winter to establish annual occupancy. We delineated territory boundaries from radio‐telemetry data using fixed kernel density estimation to create a 3D utilization distribution (UD). Each kernel's bandwidth was established using the “plug‐in” method (Gitzen, Millspaugh, & Kernohan, 2006; Wand & Jones, 1995) with the “adehabitatHR” and “ks” libraries in R 3.2.2 (Calenge, 2006; Duong, 2007; R Core Team, 2015). We used a minimum of 30 locations after removing outliers (≥5 km from territory; Fuller, 1989) and defined the annual pack territory as the 95% volume isopleth boundary (Seaman et al., 1999; Uboni, Vucetich, Stahler, & Smith, 2015). For years and/or packs with inadequate locations for estimation of the UD, we approximated territory boundaries by combining long‐term telemetry locations associated with known packs and locations of tracks from the winter tracking survey. In cases where telemetry locations did not exist we used a minimum convex polygon from long‐term track locations.

We analyzed spatiotemporal variation in wolf density by generating a longitudinal matrix representing pack persistence and changes in pack size over time. These data were linked to the pack territory boundaries in ArcMap 10.3.1 (Environmental Systems Research Institute, Inc., Redlands, CA, USA). Annual pack counts were converted to wolves/1000 km^2^. We generated a smoothed surface for annual wolf density each year using a circular moving window with radius equal to approximate median wolf dispersal in the Great Lakes region (~38 km; Treves, Martin, Wiedenhoeft, & Wydeven, 2009).

### 2.3 Landscape covariates

We developed habitat metrics representing variability in land cover, topography, prey availability, and risk of human conflict using publicly available GIS data (Table 1). We used moving window analyses to develop spatially explicit surfaces for each landscape feature considered. The initial spatial scale for each landscape feature was a 30 × 30 m cell size, which corresponded to the spatial resolution of National Land Cover Database (https://www.mrlc.gov/) and Digital Elevation Model (https://nationalmap.gov/elevation.html) products. We set the circular moving window to 50.75 km^2^ (1/4 of the mean wolf home range size), which was chosen to represent within‐territory level variation. Each 30 m cell in the resulting surface thus represented the neighborhood mean (continuous input data such as elevation) or percentage of landscape (binary input data such as land cover type) occurring within ~4.02 km of a given location on the landscape. We updated each wolf observation with habitat metrics and regional wolf density corresponding to location *i* at time *t*, thus representing the effect of “third‐order” or location‐based habitat selection (DeCesare et al., 2012; Johnson, 1980).

**Table 1:** List of codes and descriptions for all variables considered in Cox Proportional Hazards models of wolf survival times in Michigan, USA, 1992–2013

Parameter	Variable type	Description and coding
Measured at capture		
Age	Continuous	Age in years, estimated at trap or updated later via necropsy info
Sex	Categorical factor	Male, female
Capture type	Categorical factor	Research, incidental
Vaccine	Indicator	1* = *received vaccination, 0 otherwise
Ivomec	Indicator	1* = *received ivomec, 0 otherwise
Weight	Continuous	Weight at capture (kg)
Time‐dependent		
Capture effects		
Translocation^a^	Indicator	1* = *translocated, 0 otherwise
Depredation^a^	Indicator	1* = *depredation event, 0 otherwise
Movement and transience		
Pack membership^a^^,^ c	Categorical factor	0* = *resident pack, 1* = *transient
Distance (transient)	Continuous	Log‐transformed distance from center of all observations
Distance (resident pack)	Continuous	Log‐transformed distance from center of territory
Movement velocity	Continuous	Log‐transformed distance between current and last observation
Habitat		
Buck kill index	Continuous	Bucks killed by hunters per km^2^, measured within moving window
% Deer wintering complex (DWC)	Continuous	Proportion of deer winter habitat within moving window
Distance to DWC	Continuous	Distance to nearest deer wintering complex within moving window
Road density	Continuous	Road density (km/km^2^) within moving window
% Impervious surface	Continuous	Developed impervious surface % of landscape within moving window
% Agriculture	Continuous	Agriculture % of landscape within moving window
% Protected Land	Continuous	Public/protected % of landscape within moving window
Snow depth	Continuous	Long‐term average of snow depth, 1 km spatial resolution^b^
Elevation	Continuous	Average elevation (m) within moving window
Slope	Continuous	Average degrees slope within moving window
Forested:Open Edge Density	Continuous	Density of forested versus open habitat edge (km/km^2^) within moving window
Stream density	Continuous	Stream density (km/km^2^) within moving window
Density dependence and time		
Wolf density^c^	Continuous	Average annual wolf density within moving window (38 km buffer^d^)
Biological year^c^	Continuous	Nonlinear effect of biological year
Day of year^c^	Continuous	Nonlinear effect of julian date (day of year)
Age^c^	Continuous	Nonlinear effect of age over time, starting with estimated age at capture

a Indicator switches from 0 to 1 at the time of the event and remains 1 afterward.

Pack membership determined by association with known pack territory and homing movement behavior.

b Snow Data Assimilation System (SNODAS; https://nsidc.org/data/g02158).

c Nonlinear effect; modeled using cubic spline function.

d Approximate median wolf dispersal distance based on distances reported in Treves et al. (2009).

### 2.4 Survival analysis

We used extended Cox Proportional Hazards (CPH) models (Benson, Patterson, & Mahoney, 2014; Smith et al., 2010; Therneau & Grambsch, 2000) to estimate wolf annual survival and test for effects of individual‐level variation, management, habitat, density, and movement on the risk of mortality. Specifically, we were interested in the survival function *S*(*t*) = *P*(*T* ≥ *t*), where *T* is the random variable representing survival time in days (Murray, 2006). To accommodate covariates in our model, we estimated the hazard function:

(1) $$h\left(t,x,\beta \right)=h_{0}\left(t\right)r\left(x,\beta \right),$$

where the overall hazard is a function of the nonparametric baseline hazard *h*_0_(*t*) and the regression risk function *r*(*x*, β) = exp (**Xβ**) =exp (*x*_1_β_1_ + *x*_2_β_2_ + ··· + *x*_*k*_β_*k*_) (DeCesare et al., 2014; Hosmer, Lemeshow, & May, 2008; Murray, 2006). Modeling the hazard according to this formulation allows for convenient and familiar interpretation of covariate effects, where the coefficients **β** indicate relative effects on the resulting hazard ratio. Subsequently, *S*(*t*) can be determined provided the hazard function is known (Murray, 2006). We assumed no parametric distributional assumption on the hazard, while the hazard ratio was assumed constant (Klein & Moeschberger, 2005; Therneau & Grambsch, 2000). However, the model can easily be extended to cases where predictors vary with time (Fieberg & DelGiudice, 2009; Fox, 2002).

We specified CPH models where the hazard was modeled according to individual, at capture factors, time‐varying age, habitat, movement, density, and time covariates (Table 1). The event of interest was the known death of the individual wolf and the time‐to‐event interval began after the first capture. When fate was undetermined, we right‐censored individuals at their last known location and time. Individuals that left the study area and were recovered dead elsewhere were also right censored. We specified a time‐dependent age covariate, where age was modeled as a smoothed function of time after capture (Fieberg & DelGiudice, 2009; Moore, 2016; Therneau & Grambsch, 2000). During the early recovery phase (pre‐2004), most wolves received vaccinations for leptospirosis, canine distemper, and parvovirus and treatment for sarcoptic mange if necessary. Wolves involved in depredation incidents were translocated during 1998–2002. A dummy indicator variable (0/1) was included for “Vaccine” and “Ivomec.” Similar indicator variables were considered for “Translocation,” “Depredation,” “Recaptured” (i.e., trapped on > 1 occasion), and “Capture Type” (researcher vs. incidentally trapped; Table 1).

We allowed habitat variables to represent instantaneous risk, with each covariate representing surrounding habitat within the 50.75 km^2^ neighborhood at location *i*, time *t*. In addition, we quantified pack membership versus transience. Pack membership was based on consistent observation within known pack territory boundaries. Transient status was assigned when individuals left a known territory and did not return (Smith et al., 2010) or were never observed occupying a territory consistently. We referred to these individuals as transients rather than dispersers, because dispersal implies permanently leaving a natal territory (Boyd & Pletscher, 1999), which was not always known. For pack status, we quantified risk associated with exploratory movements by calculating the distance from each observation to the center of the pack's home range. We also computed a variable indicating average movement velocity corresponding to the log‐transformed distance between the current and last location, corrected for the time interval between observations. In the CPH model, we specified log‐transformed distance from the pack territory center as an interaction conditional on pack membership; for transients, the distance was calculated based on the geographic center of all observations for the individual. Movement variables were best described as orders of magnitude on the log‐scale (e.g., Ovaskainen et al., 2008) because large carnivore movements can vary widely depending on whether individuals are making territorial versus exploratory or transient movements. To model long‐ and short‐term trends and density‐dependent survival, we included variables for wolf density, day of year (DOY), and biological year. Each of these predictors was specified to have a nonlinear functional relationship with the hazard, which we accommodated using cubic smoothing splines with three initial degrees of freedom (Harrell, 2015; Moore, 2016; Therneau & Grambsch, 2000).

We specified a full model by initially including all parameters (Table 1) and then used Lasso regularization techniques to screen against overfitting by shrinking redundant and/or unimportant predictors to 0 (Simon, Friedman, Hastie, & Tibshirani, 2011; Tibshirani, 1996). To implement the Lasso, we used the elastic net regularization penalty via “glmnet” in R, setting the parameter α to 1.0 (Friedman, Hastie, & Tibshirani, 2010; Tibshirani et al., 2012). To determine which effects to keep, we allocated a minimum of 15 degrees of freedom to each model parameter, such that the ratio of sample size (*n = *number of observed mortality events) to predictor degrees of freedom (*k)* did not exceed 15 (Harrell, 2015). As such, the initial Lasso procedure produced a model with *k* predictor degrees of freedom. The Lasso did not automatically select all knots associated with a nonlinear covariate (e.g., approximated by cubic splines), so if any nonlinear effect was selected, we included all knots associated with that term in a subsequent model fit. We refitted the resulting model with a cluster term for each wolf pack to account for within‐pack correlation using robust variance estimation (Therneau, 2016; Therneau & Grambsch, 2000). If *n/k* exceeded 15 at this point, we proceeded with model reduction according to Harrell (2015, pp. 521–525) by dropping any remaining unnecessary or redundant predictors. To check the assumption of proportional hazards for the final model, we plotted scaled Schoenfeld residuals over time for each covariate and tested for a statistically significant trend (β(*t*) ≠ 0; DeCesare et al., 2014; Moore, 2016). We fit all models using the “survival” and “rms” packages in R 3.2.2 (Harrell, 2015; R Core Team, 2015; Therneau & Grambsch, 2000), with Lasso model selection implemented in “glmnet” (Friedman et al., 2010; Simon et al., 2011).

### 2.5 Risk maps

We used our final model to predict spatial representations of annual survival for three time periods (and population sizes) during the study: early recovery (1995–2000; <250 wolves), mid‐recovery (2001–2006; 250–450 wolves), and late recovery (2007–2013; 450–700 wolves). To obtain estimates, we conditioned on the average (for continuous variables) or most common case (non‐spatial variables), and projected cumulative annual survival estimates onto a map of the study area using the local neighborhood estimates for relevant landscape predictors in the model formula:

(2) $${\hat{S}}_{i}\left(t\right)={\hat{S}}_{0}{\left(t\right)}^{\exp(x_{i}^{\prime }\hat{\beta })},$$

where ${\hat{S}}_{i}\left(t\right)$ represents expected survival probability for an “average” individual at location *i* and time *t* (365 days in this case), and ${\hat{S}}_{0}\left(t\right)=\text{exp}\left(-{\hat{\Lambda }}_{0}\left(t\right)\right)=\text{exp}\left(\Sigma_{j:t_{j}\leq t}{\hat{h}}_{0}\left(t_{j}\right)\right)$ (DeCesare et al., 2014; Fieberg & DelGiudice, 2009; Therneau & Grambsch, 2000). For each time period, we estimated $\hat{S}\left(t\right)$ separately for males and females with initial age set to 3 years, and specified relevant time‐dependent covariates such that their paths could be mapped through time for each prediction (Thomas & Reyes, 2014). For the final risk maps, we averaged all survival probabilities for males and females to represent the population‐level estimate.

## 3 RESULTS

The MDNR monitoring program produced 365 individual wolf encounter histories, with 176 known deaths occurring during the study. The remaining individuals were right‐censored, either because fate was undetermined or because they were retrieved dead later outside of the study area. The estimated overall annual survival rate for collared wolves during our study was 0.75 (95% CI* = *0.70–0.80), which was similar to other U.S. populations with estimates ranging from 0.75 (Smith et al., 2010; Wydeven et al., 2009) to 0.79 (Adams et al., 2008; Cubaynes et al., 2014; Wydeven et al., 2009). CPH models fit with covariates revealed that multiple factors influenced mortality risk and subsequent survival estimates. Variable screening and model reduction resulted in a model with *k = *12 predictor degrees of freedom. The best‐reduced model included capture‐level covariates for sex, capture type (research vs. incidental), and translocation status (Table 2). In addition, time‐dependent covariates were supported for pack membership versus transience, age (initial age + time after capture), DOY, wolf density, forested‐open edge density, and proportion of agriculture (Table 2). Nonlinear effects were supported for age, DOY and wolf density, although the number of knots was ultimately reduced to 2 for each spline term to avoid overfitting models to noise in the data. The proportional hazard assumption was satisfied for all predictors (Table 3).

**Table 2:** Relative effects (log hazard) of relevant predictors in a selected Cox Proportional Hazards model of wolf survival times in Michigan, USA, 1992–2013. Negative values correspond to reduced mortality risk

Parameter	$\hat{\beta }$	*SE* ($\hat{\beta }$)	Wald *Z*	*p*
Sex				
Male	0.355	0.156	2.280	.023
Capture effects				
Weight at capture				
Translocation	−0.467	0.340	−1.370	.170
Depredation				
Recaptured				
Researcher (vs. Incidental)	−0.238	0.201	−1.19	.235
Vaccine				
Ivomec				
Movement and transience				
Territory membership (1* = *resident)	−1.45	0.213	−6.850	<.001
Distance from center of observations (transient)				
Distance from territory (territory occupant)				
Movement rate				
Habitat				
Buck kill index				
% Deer wintering complex				
Distance to deer wintering complex				
Road density				
% Impervious surface				
% Agriculture	0.102	0.066	1.540	.125
% Protected land				
Snow depth				
Elevation				
Slope				
Forested:Open Edge Density	0.217	0.083	2.610	.009
Stream density				
Density dependence and time				
Age_1_	−0.207	0.102	−2.03	.043
Age_2_	0.187	0.125	1.50	.134
Day of year (DOY)_1_	−0.007	0.002	−3.26	.001
DOY_2_	0.008	0.002	3.31	.001
Biological year, linear term				
Biological year, nonlinear terms				
Wolf density_1_	0.701	0.265	2.64	.008
Wolf density_2_	−0.843	0.442	−1.91	.056

**Table 3:** Results of the assumption of proportional hazards test using scaled Schoenfeld residuals for each individual predictor separately and for the full (global) model, where *p* < .05 indicates a statistically significant relationship between a predictor's effect and time

Parameter	ρ	χ^2^	*p*
Age	−0.009	0.020	.889
Sex	−0.017	0.063	.801
Translocation	0.014	0.047	.829
Agriculture	0.010	0.029	.865
Territory membership	−0.034	0.219	.640
Wolf density	−0.109	2.465	.116
Day of year	−0.014	0.037	.847
Edge	−0.021	0.082	.778
Global	NA	2.906	.968

**Figure 1:**
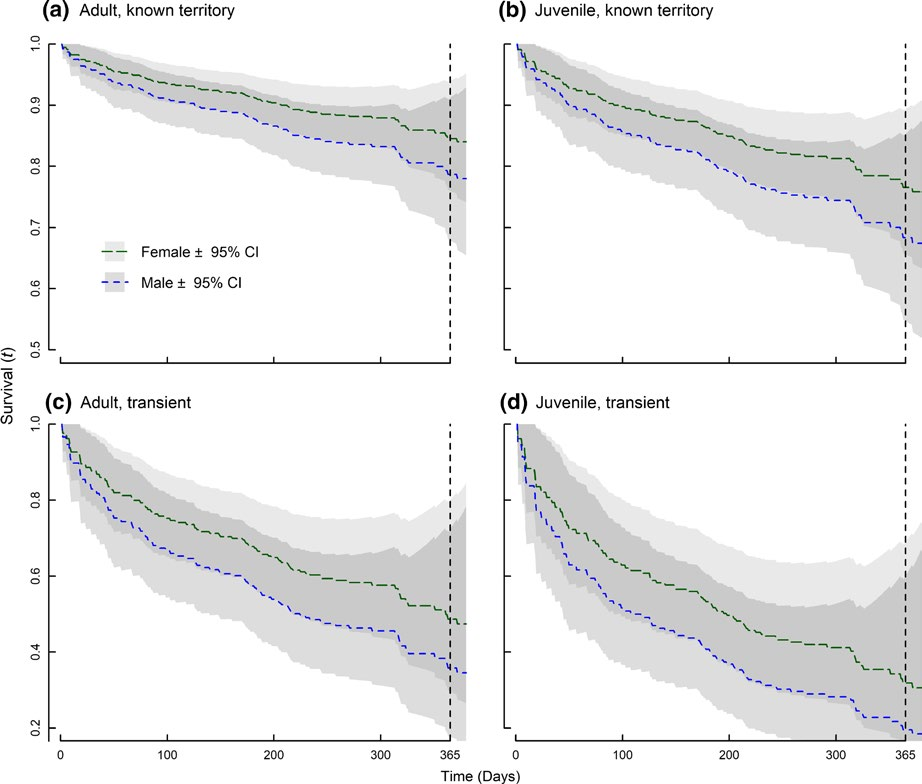
Predicted annual survival rates from a Cox Proportional Hazards (CPH) model comparing adult and juvenile wolves occupying territories (a, b) to adult and juvenile transient wolves (c, d) in Michigan, USA, 1992–2013. Females (green curves) had greater survival rates than males (blue curves), and survival varied seasonally based on a smoothed function of time (Julian day) with mortality risk greater in winter than in summer. Transient status was based on movements away from known territories without returning and had lower predicted survival (c, d). Initial age was 1 year old for juveniles and 3.5 years for adults; all other covariates in the CPH were held constant at mean values for continuous variables or most common case for discrete or factor variables

**Figure 2:**
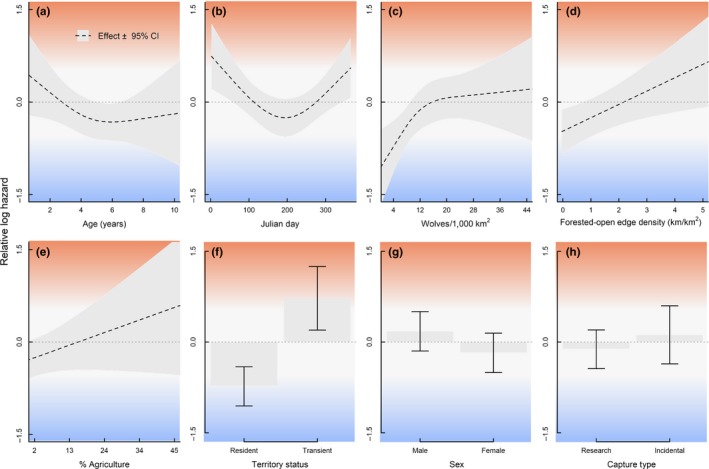
Relative log hazard effects from a Cox Proportional Hazards fit to time‐dependent predictors in Michigan USA, 1992–2013. Greater log hazard indicates greater mortality risk and shorter survival times (red) while lower hazards correspond to lower risk and longer survival times (blue) for a) Experience b) Seasonality c) Density‐dependence d) Forest edge e) Agriculture f) Territory g) Sex h) Capture

Mortality risk was ~1.43 times greater for male wolves (${\hat{\beta }}_{\mathrm{male}}$ *= *0.355, *SE = *0.156, *p = *.023) and decreased when individuals were associated with a known pack territory (${\hat{\beta }}_{\mathrm{resident}}$ *= *−1.455, *SE = *0.213, *p* < .001; Figure 1). Mortality risk was also reduced as individuals aged and gained experience through about ~5 years of age before stabilizing and slightly increasing thereafter (Figure 2; ${\hat{\beta }}_{{\mathrm{age}}_{1}}$ *= *−2.065, *SE = *0.102, *p = *.043; ${\hat{\beta }}_{{\mathrm{age}}_{2}}$ *= *0.187, *SE = *0.125, *p = *.043). Twenty‐four wolves were translocated following depredation events; our model indicated that this action may have reduced their mortality risk (${\hat{\beta }}_{\mathrm{translocated}}$ *= *−0.467, *SE = *0.34, *p = *.167). In addition, our model indicated that incidentally trapped wolves had greater mortality risk than research‐trapped wolves, although the effect size was relatively weak (${\hat{\beta }}_{\mathrm{research}}$ *= *−0.238, *SE = *0.201, *p = *.235).

Landscape covariates representing prey availability, land cover, topography, and risk of human‐caused mortality had relatively little effect overall on mortality risk, with only two of the original 12 landscape predictors retained in the final model (forested‐open edge density, % agriculture; Table 2). Risk increased with greater proportions of agriculture (${\hat{\beta }}_{\mathrm{agriculture}}$ *= *0.102, *SE = *0.066, *p = *.125) and with increasing edge densities (${\hat{\beta }}_{\mathrm{edge}}$ *= *0.217, *SE = *0.083, *p = *.009).

**Figure 3:**
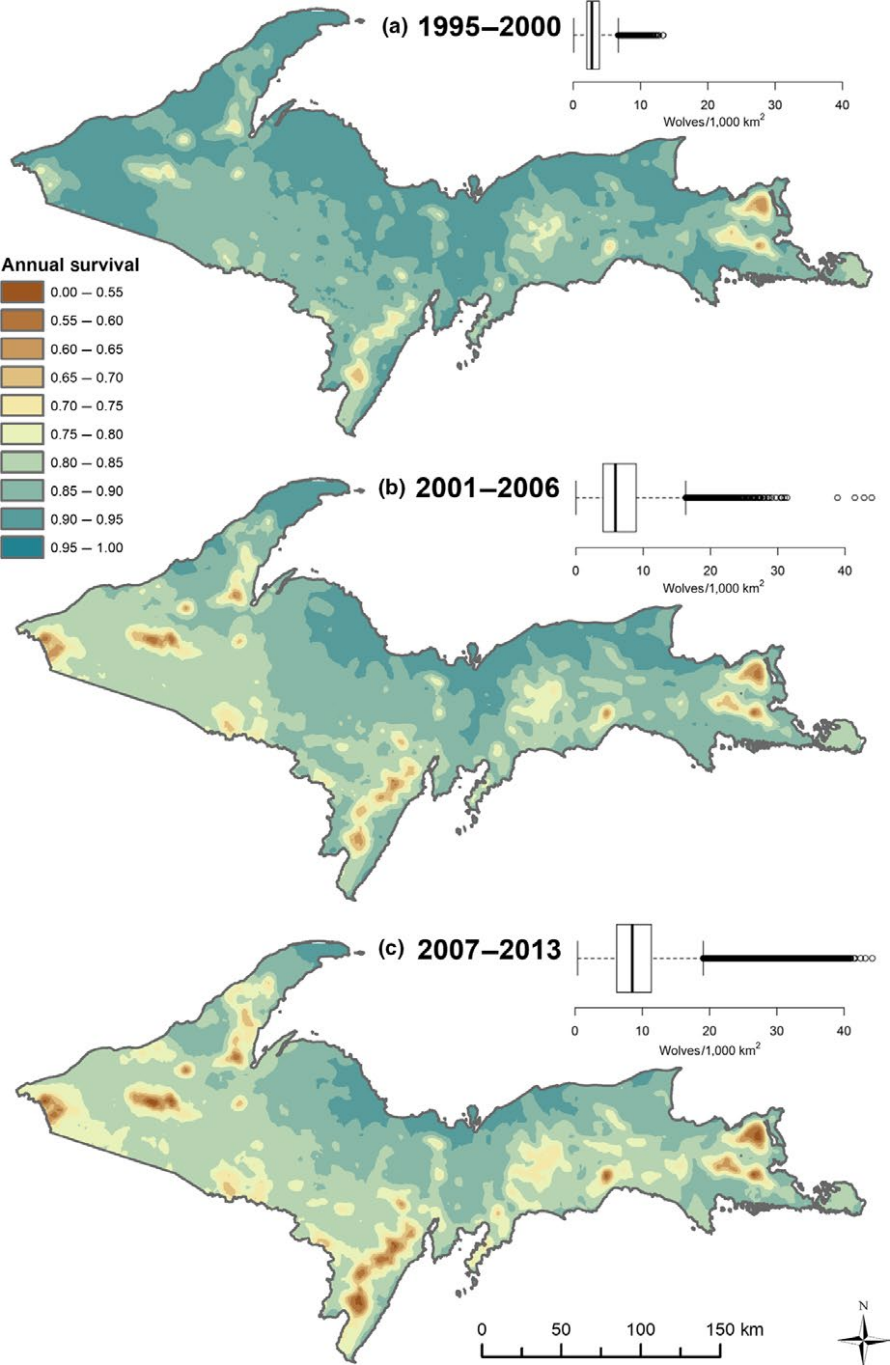
Spatial representation of the landscape of risk for wolves in Michigan, USA corresponding to three time periods: (a) 1995–2000; early recovery, (b) 2001–2006 (mid‐recovery), and (c) 2007–2013 (late recovery). Spatial and temporal variation in predicted survival reflected density dependence (lower survival rates with increasing wolf density), and landscape effects associated with agriculture and open versus forested edge densities (increased mortality risk with greater proportions of agriculture and greater edge densities). Annual survival estimates were for adult wolves (starting age* = *3.5 years), and estimates were conditioned on the 1st day of the biological year (April 15)

We detected seasonal (DOY) and density‐dependent effects on mortality risk, which had nonlinear effects on the hazard (Table 2; Figure 2). Mortality risk was greatest in winter and lowest in summer (${\hat{\beta }}_{{\mathrm{doy}}_{1}}$ *= *−0.007, *SE = *0.002, *p = *.001, ${\hat{\beta }}_{{\mathrm{doy}}_{2}}$ *= *0.008, *SE = *0.002, *p = *.001; Figure 2b). Survival was density‐dependent (${\hat{\beta }}_{{\mathrm{density}}_{1}}$ *= *0.701, *SE = *0.265, *p = *.008; ${\hat{\beta }}_{{\mathrm{density}}_{2}}$ *= *−0.843, *SE = *0.442, *p = *.056, with the estimated hazard increasing with greater wolf densities; the increase was sharp initially before apparently stabilizing at moderate densities (Figure 2c). Density dependence associated with spatiotemporal variation in wolf density was reflected by our risk maps, as estimated survival rates declined the most in the highest wolf density areas over time (Figure 3).

We contrasted predicted survival rates across wolf demographics in our study to demonstrate the magnitude of effects in our final models. Wolves in the study were typically captured by a researcher and never translocated; assuming these conditions and average values for environmental predictors (forested vs. open edge density, % agriculture, wolf density), expected annual survival was 0.85 (95% CI* = *0.76–0.94) for adult females and 0.79 (0.68–0.91) for adult males (Figure 1a,c). In contrast, less than half of transient yearling individuals were expected to survive a year on average (assuming age* = *1 at capture; *S = *0.32 [0.15–0.69] and *S = *0.20 [0.06–0.60] for females and males, respectively; Figure 1b,d). Adult wolves occupying risky habitats with high wolf densities (edge density, % agriculture and wolf density all occurring at 90th percentiles) had reduced survival (*S = *0.73 [0.59–0.91] and *S = *0.64 [0.47–0.64] for males and females, respectively).

## 4 DISCUSSION

Annual survival is a key vital rate affecting population dynamics in large carnivores and provides important information about how preferred habitats influence fitness. Linking habitat to fitness metrics such as survival is likely to be more valuable for long‐term management and conservation of populations than focusing solely on habitat selection or species distribution (Franklin, Anderson, Gutiérrez, & Burnham, 2000; Gaillard et al., 2010), especially when species’ deviate from the theoretical ideal‐free habitat distribution (Mosser et al., 2009). Our models related patterns in wolf movement, territory use, and density dependence to spatiotemporal variation in survival, reinforcing that the spatial ecology of this species is a key component of long‐term fitness and population trends. By identifying the most relevant predictors of survival and mortality risk, we can extrapolate predictions of a key fitness indicator spatially and temporally, providing a valuable tool for effective management of a controversial but ecologically important top predator. From the results of our analysis, we identified four areas of focus that are broadly relevant to large carnivores under the context of spatiotemporal variation in survival and mortality risk: (1) long‐term temporal variation and density dependence in estimates of survival, (2) short‐term seasonal variation and its ecological relevance and potential management implications, (3) human impacts and the importance of navigating the anthropogenic landscape of risk (Stenglein, Gilbert, Wydeven, & Van Deelen, 2015), and (4) the importance of identifying habitat quality based on fitness indicators for despotic or preemptive habitat selectors (Mosser et al., 2009; Pulliam & Danielson, 1991).

**Figure 4:**
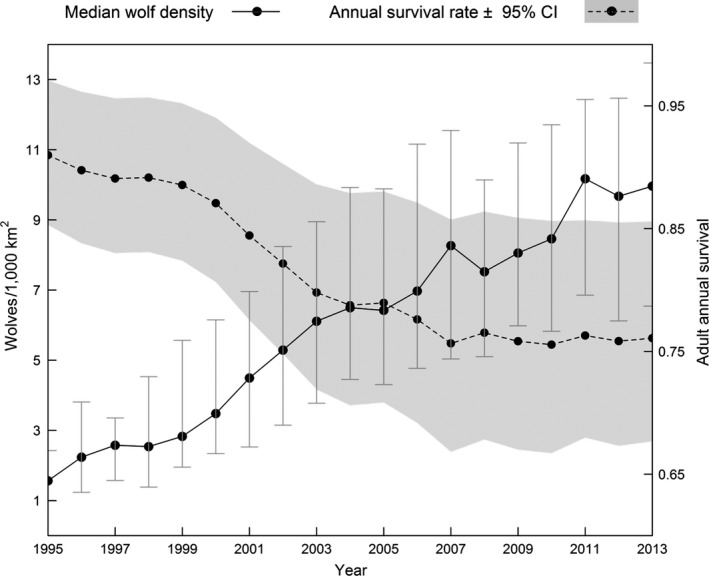
Time trend in annual survival rates for an average adult wolf corresponding with changes in median wolf density in Michigan, USA, 1995–2013. Wolf abundance increased from 57 to >600 during the study; declines in survival were related to increasing wolf density, as survival predictions were obtained from a Cox Proportional Hazards model with all predictors except wolf density held constant at their average (continuous variables) or most common values (factor variables) in the study. Error bars around the density estimates represent the interquartile range, while the shaded polygon around the survival estimates represents the 95% confidence interval

Density dependence was a driving force of long‐term temporal variation in survival rates. We included smoothed terms for time (biological year) in addition to wolf density, but only spatiotemporal density was retained as an important predictor (Table 2). The effect was evident in spatial predictions of annual survival during early (1995–2000), mid (2001–2006), and late (2007–2013) recovery time periods (Figure 3). When holding all other variables constant in our model, estimated survival rates declined as a function of density across the time series (Figure 4). By the late time period, modeled survival was lowered throughout the majority of the study area (Figure 3c), which we attributed mainly to broad‐scale increases in wolf density because proportions of agriculture and forested‐open edge densities were reasonably constant while wolf density varied over time (Figure 4). Density‐dependent regulation of survival rates in wolves can occur through increased intraspecific aggression when wolves are protected from human‐caused mortality (Cubaynes et al., 2014). Although we documented a few cases of wolves being killed by other wolves (*n = *9; MDNR unpublished data)*,* we observed more evidence that the declines in survival in our study area corresponded with increased potential for human conflict. As wolves expanded their range, shifts in habitat use and selection resulted in greater exposure to suboptimal habitat with greater proportions of agriculture and human development, subsequently increasing the risk of human‐caused mortality due to legal lethal control, poaching, or vehicle strikes.

We detected seasonal variation in the hazard rate for wolves in our study. Mortality risk increased during fall and winter and appeared to peak in January (Figure 2c). Wolf kill rates of deer were observed to be lower during December–mid‐February than later in winter (mid‐February–April) (Vucetich et al., 2012), indicating that difficulty capturing prey could contribute to reduced survival. Wolves may also be more vulnerable to illegal human‐caused mortality during winter. Deer hunting with firearms in our study area typically began in mid‐November, with a muzzleloader season concluding in mid‐December. Illegal killing likely occurs opportunistically in heavily forested regions; wolves are probably most vulnerable to being killed illegally during hunting seasons due to the increased probability of encounters with humans with firearms. However, perceptions that large carnivores have contributed to reduced populations of game species may also play a role, particularly when hunter satisfaction is low (Treves, Naughton‐Treves, & Shelley, 2013). Seasonality in mortality risk also coincides with the heaviest snowfall time periods in the UP, where recreational trails receive heavy snowmobile use. Wolves are known to use forest roads and trails as travel routes and territory boundaries (Kohn, Anderson, & Thiel, 2009; Whittington, St Clair, & Mercer, 2005), which likely makes them more vulnerable to detection (Zimmermann, Nelson, Wabakken, Sand, & Liberg, 2014). Alternatively, dispersal may vary seasonally in wolf populations (Kojola et al., 2006) and transience was associated with increased mortality risk in our study, indicating that dispersal could contribute to seasonal variation in mortality risk.

Drivers of mortality risk suggested that human impacts were the predominant hazard facing wolves, even in a population that was legally protected during the majority of the study (Beyer et al., 2009; Olson et al., 2015). Mortality was primarily human‐caused, with the majority of deaths occurring due to poaching, vehicle strikes, and other human causes such as legal euthanization or incidental trapping (O'Neil, 2017). Records of known mortality sources indicated human mortality causes outnumbered other causes by >2:1. Human‐caused mortality tends to be prevalent among large carnivore species (Ripple et al., 2014) suggesting that our findings of density‐dependent increases in mortality risk for wolves are likely generalizable across other large carnivores. Given that large carnivore populations are expanding in many areas, conflict rates are also likely to increase. Local or regional density‐dependent increases in mortality risk may occur in other predator species as well, as distributions begin to overlap more developed landscapes. Although large carnivore species may adapt to novel environments which allows niche shifts to occur (Moss, Alldredge, Logan, & Pauli, 2016; Moss, Alldredge, & Pauli, 2016; Smith, Wang, & Wilmers, 2016), this likely comes at the cost of reduced survival and increased anthropogenic mortality (Moss, Alldredge, & Pauli, 2016). In such situations, population growth and continued range expansion for these species will likely depend on human tolerance and protections, compensatory mechanisms, and productive source populations that promote population growth in the presence of locally high‐mortality rates (Chapron et al., 2014; Linnell, Swenson, & Anderson, 2001; Weaver, Paquet, & Ruggiero, 1996).

Our final survival model included effects of proportion of agriculture, open‐forest edge density, territory versus transient, translocation, and experience, which suggests that occupying high quality “real estate” (Mosser et al., 2009), developing knowledge of territory, and learning to navigate a risky landscape are keys to long‐term survival for wolves and other large carnivores. Evidence for this includes the following: first, experience mattered, as risk generally decreased as wolves got older and transients were more likely to survive as they aged and established territories. Our model suggested that the optimal age for a wolf in terms of experience was between 4 and 6 years old, after which mortality risk increased due to senescence (Figure 2a). Second, density dependence in survival rates combined with habitat predictors indicative of risk (Figure 2c–e) suggests potential for a source‐sink process consistent with ideal‐despotic or ideal‐preemptive habitat distributions (Morris, 2003; Mosser et al., 2009; Pulliam & Danielson, 1991). According to this scenario, early colonizers occupy the safest habitat and later colonizers must choose from riskier sites. Consequently, per capita mortality risk increases with density as some individuals are forced into marginal habitats with greater exposure to human conflict, as characterized by farms and fragmented landscapes. The ideal‐despotic model would thus predict uneven fitness occurring across the landscape, where high quality habitats (sources) contribute most to population growth, while marginal habitats have a net‐negative contribution (sinks; Pulliam & Danielson, 1991; Mosser et al., 2009). Our results provide support for the possible development of a source‐sink structured population by demonstrating density‐dependent, spatial variation in survival (Figure 3).

Source‐sink dynamics are complex, and demonstration of source versus sink habitat would require additional information on recruitment and immigration/emigration to allow estimation of a net growth rate for specific habitats (Furrer & Pasinelli, 2016). Thus, spatial predictions of annual survival should be interpreted carefully, with greater risk indicating conflict areas that may occasionally function as sinks (e.g., Ruth et al., 2011; Smith et al., 2010). Alternatively, when carnivores are prone to conflicts with humans and anthropogenic mortality risk is high, then density‐dependent increases in mortality risk may simply be a function of carnivore–human encounter rates. In this case, increased conflict rates co‐occur with carnivore population expansion because the probability of encounter increases (Gurarie & Ovaskainen, 2013; Hutchinson & Waser, 2007). In our study, this is corroborated by greater survival rates predicted for wolves that consistently occupy territories as opposed to increased hazards for those that exhibit exploratory movements and transience. Population increase thus results in greater abundance of transient, mobile individuals which are more likely to have risky encounters with humans, thereby reducing survival.

## 5 CONCLUSION AND RECOMMENDATIONS

Large carnivores may preemptively colonize high quality habitats (O'Neil, 2017), leading to site‐dependent population regulation (Mosser et al., 2009; Rodenhouse, Sherry, & Holmes, 1997). Preemptive site occupancy and site‐dependent population dynamics can result in uneven fitness and increased potential for human conflict across landscapes when high quality habitat becomes saturated (Murray et al., 2010; Smith et al., 2010). Declining survival rates in wolves were related to spatiotemporal variation in wolf density during recolonization, indicating that mortality risk is density‐dependent when safe habitats are limited. In this case, a mismatch between habitat suitability and occupancy may exist, and traditional habitat suitability analyses may be unreliable indicators of quality. Source‐sink population dynamics may occur in this scenario, but potential conflict areas may not be apparent at low densities. Quantitative ecologists and resource managers should seek to integrate spatiotemporal variation into models of habitat selection and survival, which will help to inform habitat‐related contributions to population growth and prioritize conservation areas. Identifying important source habitats and preserving them will promote and help to sustain long‐term, regional species recovery for expanding carnivore populations.

## AUTHOR CONTRIBUTIONS

STO, JKB, and DEB conceived ideas, compiled long‐term datasets, designed methodology, and contributed critically to drafts; STO analyzed the data and led writing of the manuscript; All authors gave final approval for publication.

## CONFLICT OF INTEREST

None declared.
