# Supplementary figures and images for: Spatially varying density dependence drives a shifting mosaic of survival in a recovering apex predator (Canis lupus)
Source: Ecol Evol. 2017 Oct 28;7(22):9518–30. doi: 10.1002/ece3.3463 (PMC5696399; doi:10.1002/ece3.3463)

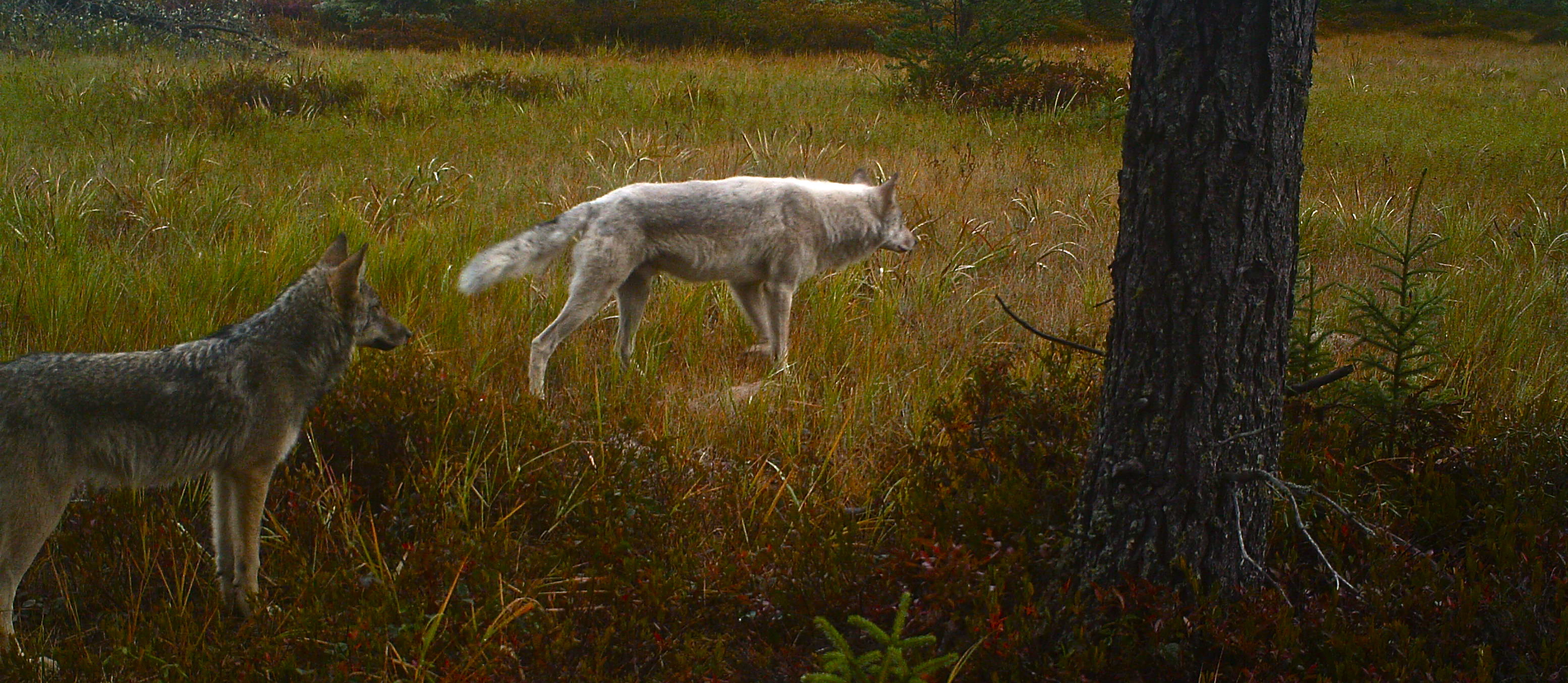

Supplement: Supplementary file 1 [file ECE3-7-9518-s001.tif]
