# Supplementary material for: Spatially varying density dependence drives a shifting mosaic of survival in a recovering apex predator (Canis lupus)
Source: Ecol Evol. 2017 Oct 28;7(22):9518–30. doi: 10.1002/ece3.3463 (PMC5696399; doi:10.1002/ece3.3463)
Supplement: Supplementary file 2 [file ECE3-7-9518-s002.pdf]

Michigan Department of Natural Resources
Wildlife Division Procedure

Estimating Wolf Abundance in Michigan

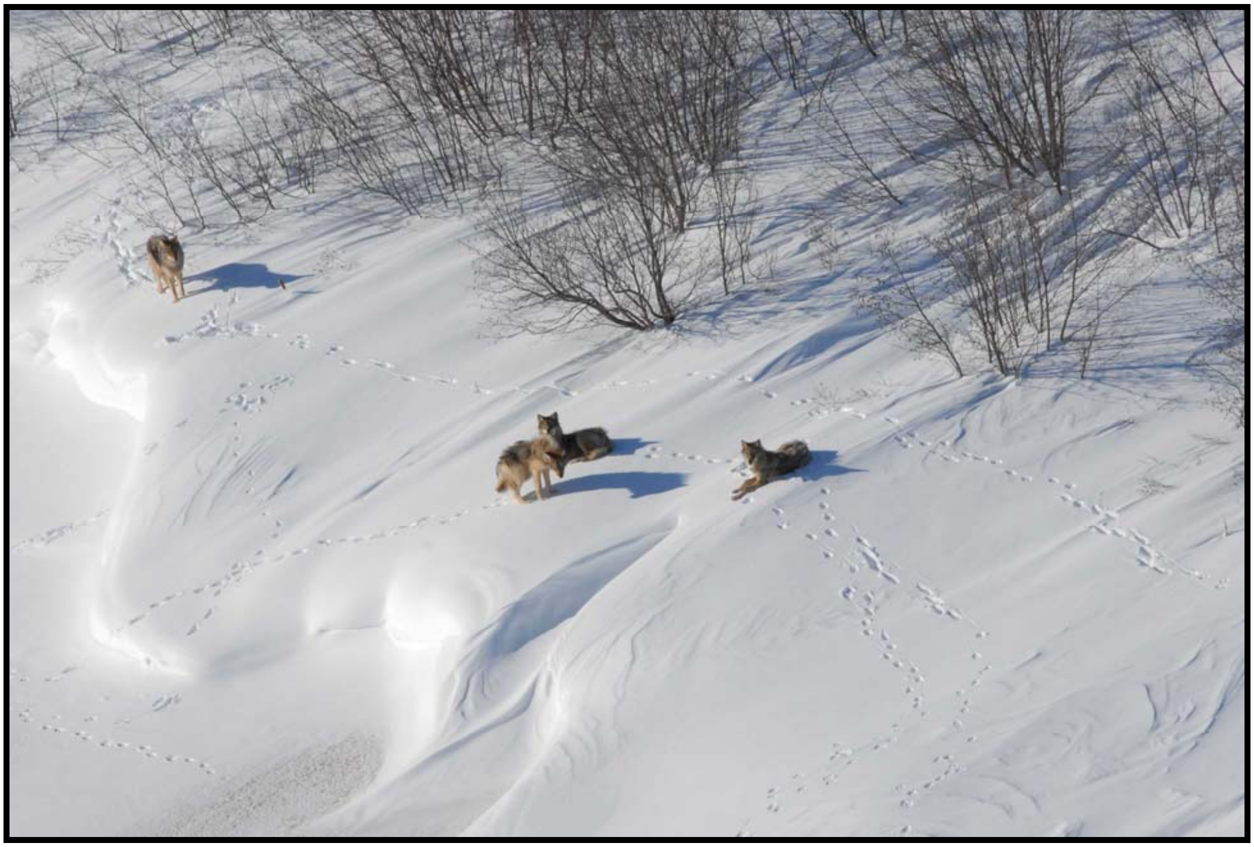

June 2008

Introduction

Estimates of abundance have been important for documenting the recovery of wolves in Michigan and the Great Lakes Region. In 2007, wolves in the Great Lakes Region were removed from the federal list of threatened and endangered species and the primary management authority for the species shifted to the states. In Michigan, wolves are still classified as a state threatened species, but the population has met state recovery criteria and delisting is anticipated. The United States Fish and Wildlife Service (USFWS) is required to monitor species for a minimum of five years after they have been removed from the federal list of threatened and endangered species. To help the USFWS fulfill this requirement and to help identify management needs, the Michigan Department of Natural Resources (DNR) has committed to monitoring wolf population size annually for five years after federal delisting. After this five-year period, the frequency and/or necessary precision of wolf abundance estimates may change depending on the type of management actions implemented or anticipated and the relative size of the wolf population.

Estimating the abundance of most species of wildlife is challenging and wolves are no exception. Wolves are cryptically colored and live in forested environment– characteristics that make them difficult to observe from the ground or air. Wolf territories are large and even at the highest densities reported (~ 40 wolves/1000 km^2; outside of Isle Royale), wolves are relatively rare on the landscape. In comparison to more-common species, such white-tailed deer (*Odocoileus virginianus*), wolf densities are very low (Figure 1).

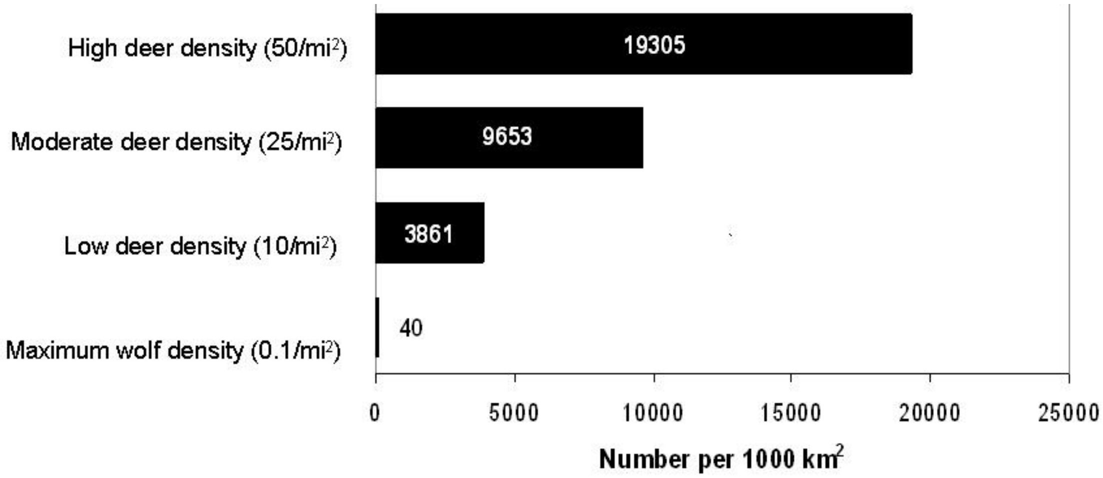

Figure 1. Comparison of a range of deer densities to an approximation of the maximum winter wolf density reported in the literature.

Wolves are firmly established in Michigan's Upper Peninsula (UP) with an estimated minimum population size of 509 animals in winter 2007. Although wolves were found in the northern Lower Peninsula in 2004, a population has not yet been established there.

Currently wolf monitoring in the UP is designed to estimate abundance and monitoring in the Lower Peninsula is designed to assess presence/absence. Because of the different objectives, different approaches are used in the two peninsulas.

Upper Peninsula

Prior to 2007, the Michigan DNR and USDA Wildlife Services estimated wolf abundance in the UP by surveying suitable wolf habitat throughout the entire peninsula during winter, when snow cover made wolves and their tracks easier to see. Surveys during winter provided an estimate of the wolf population at its smallest size in the annual cycle. Winter surveys consisted of intensive and extensive searches of roads and trails by truck and snowmobile for wolf tracks and other sign (Potvin et al. 2005). Trackers searched for wolf sign by traveling along roads and trails in trucks or on snowmobiles. Trucks and snowmobiles typically traveled at 5 to 24 km/hr (3 to15 mph) depending on snow surface conditions and traffic. When wolf tracks were found, the number of animals was estimated by following the tracks as far as practicable, searching for distinct trails of individual animals. Other information collected included the date, time, location of the tracks, measurements of track size and stride length, estimated age of the track, and the distance followed. The time since the last snowfall was recorded and used to help estimate track age. Scent-marks such as urination, defecation and scratching were recorded to help identify territorial pairs and packs. Observations of blood in urine and raised-leg urinations provided indirect evidence of breeding.

These track surveys were extensive because much of the UP, which encompasses about 43,000 km^2, is suitable habitat. An average of 12,257 km of roads and trails (~25% of available roads and trails) were searched at least once each year from 2000 through 2006. The surveys were intensive because many roads and trails had to be searched multiple times before an accurate count could be made. Searching for wolf sign was systematic and was guided by several sources of information. In the beginning, trackers used observations of wolves or wolf sign reported by citizens to help identify areas where wolves might be established. As survey data accumulated, prior results guided subsequent surveys. Perhaps the most important source of information was the movement pattern of wolves determined by radio-tracking radio-collared animals. Wolves are highly territorial and radio-collared animals provided measurements of the sizes and locations of numerous territories. Counts of packs with radio-collared members were also made through airplane observations of pack members associated with radio-collared wolves; however, heavy forest cover in the UP makes it difficult to observe wolves. In addition, packs often split into smaller hunting groups (Mech and Boitani 2003), so many flights may be necessary to find an entire pack together in an open area where it can be observed.

Wolves are very mobile, averaging movements of 14 to 28 km per day (Mech 1966; Ciucci et al. 1997; Jedrzejewski et al. 2001). These movements present a challenge for survey crews; trackers must use care when they find tracks in adjacent areas to ensure that individual wolves are not being counted more than once. Trackers avoided double-counting of wolves in adjacent areas by using the territorial boundaries of radio-collared animals to distinguish between discrete groups of wolves. In areas without radio-collared wolves, differentiation of packs depended on finding fresh tracks in adjacent areas with no sign of movement between the areas (Potvin et al. 2005).

As a result of these procedures, the reported numbers of wolves represent minimum estimates of population size.

A single, isolated set of wolf tracks could represent a lone animal or a pack member that happens to be traveling by itself. Therefore, a conservative approach was used to include lone wolves in annual population estimates. During 2001–2006, the percentage of lone wolves in abundance estimates was only 2–3%. Estimates of the proportion of lone wolves in various populations range from 7 to 20% but are generally between 10 to 15% (Fuller et al. 2003). If lone wolves represent as much as 15% of the population, Michigan's minimum population estimates underestimated actual population size by approximately 12%.

A rigorous assessment of the accuracy of wolf population estimates was conducted during a 4-year period from 2001–2005. Michigan Technological University (MTU) and the Michigan DNR conducted two independent wolf surveys in a 1,940 km^2 study area in the western UP (Huntzinger et al. 2005). No communication on survey results between the two groups was allowed until each year's survey was completed. The MTU crew spent the majority of the winter counting and recounting wolves in the study area. The DNR crew spent much less time surveying this area because it also needed to conduct surveys in the rest of the UP. Thus, it was assumed that the MTU estimates would be more accurate. Overall, the MTU and DNR counts were similar, with an average difference of only 4%. These results indicate the DNR survey results were accurate estimates of abundance.

As the wolf population increased, separating adjacent packs became more difficult and time-consuming. In 1995, the wolf population was estimated at a minimum of 80 animals distributed among 27 packs and the average distance from a pack to its nearest neighbor was 28 km (17.5 mi). By 2006, the wolf population had grown to at least 434 animals and 91 packs and the average distance from a pack to its nearest neighbor had declined more than 50% (13 km; 7.9 mi). The increased number of packs in close proximity made distinguishing them from one another difficult (Fig. 2).

Because a complete survey of the UP was becoming less practicable, a new approach to estimating wolf abundance was developed. Most commonly, a sampling approach is used when it is not possible or too expensive to survey an entire area. The complete surveys that had been conducted in the past provided an opportunity to retrospectively evaluate multiple sampling approaches. Based on these evaluations, it was determined that a geographically stratified sampling scheme would produce unbiased, precise estimates of wolf abundance (Potvin et al. 2005, T. Drummer, MTU, unpublished data). The sampling scheme developed reduces the area that needs to be searched, allowing trackers to spend more time in smaller areas. It allows trackers to search areas more thoroughly to determine whether wolves observed in adjacent areas belong to the same packs or different packs.

The UP is divided into 21 units from which a stratified random sample is drawn (Fig. 3). Twelve or 13 survey units, allocated roughly equally among strata, are selected for sampling each year. This number of units ensures that more than 50% of the UP is surveyed every year. Field personnel (trackers) are assigned responsibility for track counts in specific units. Surveys in adjacent units are coordinated in time and space along unit boundaries to avoid duplicate

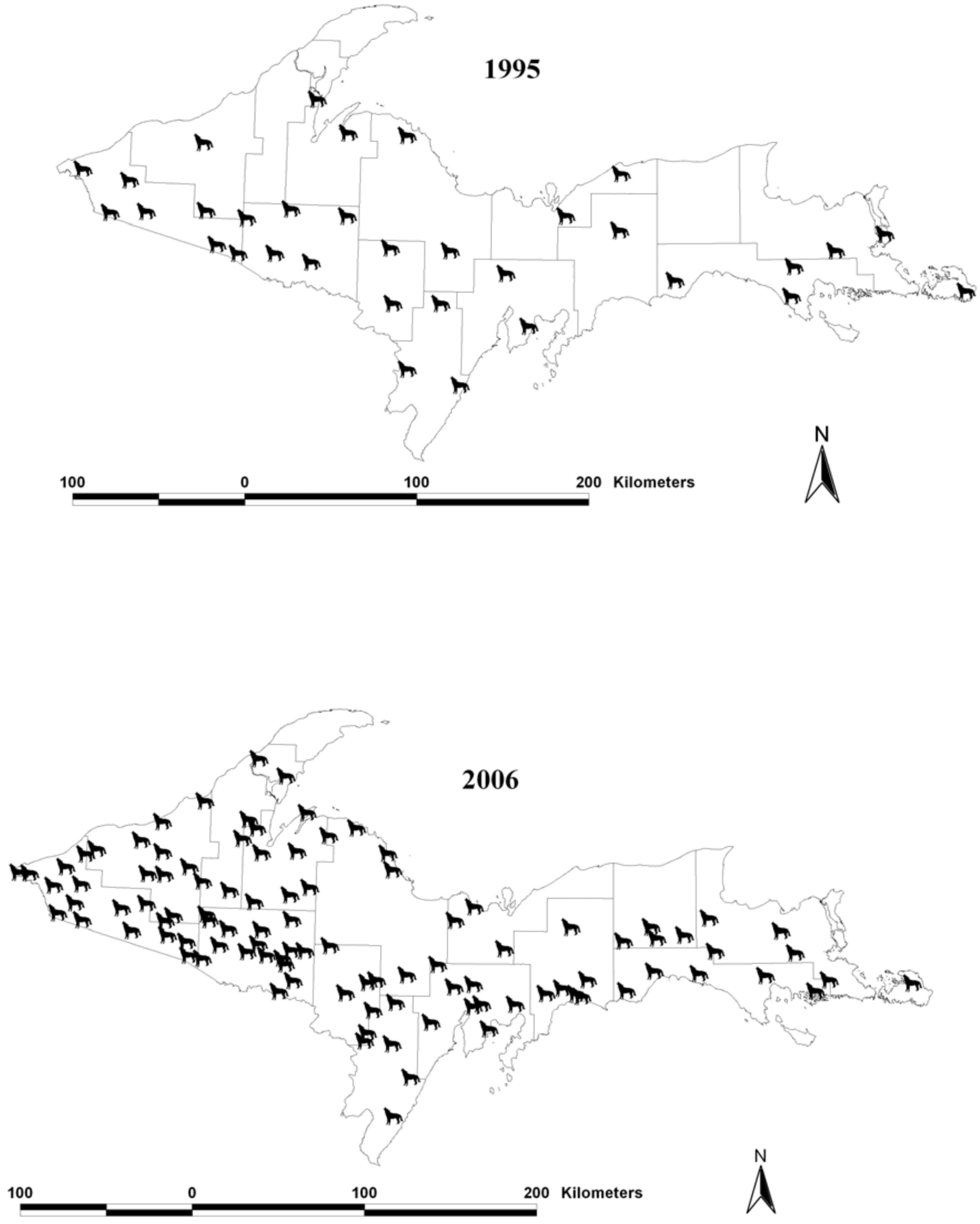

Figure 2. Distribution of wolf packs in 1995 and 2006 in Michigan's Upper Peninsula. Note the increase in the number of packs and the decrease in the average spacing between adjacent packs from 1995 to 2006. These changes resulted in the need to develop a new approach to estimating wolf abundance.

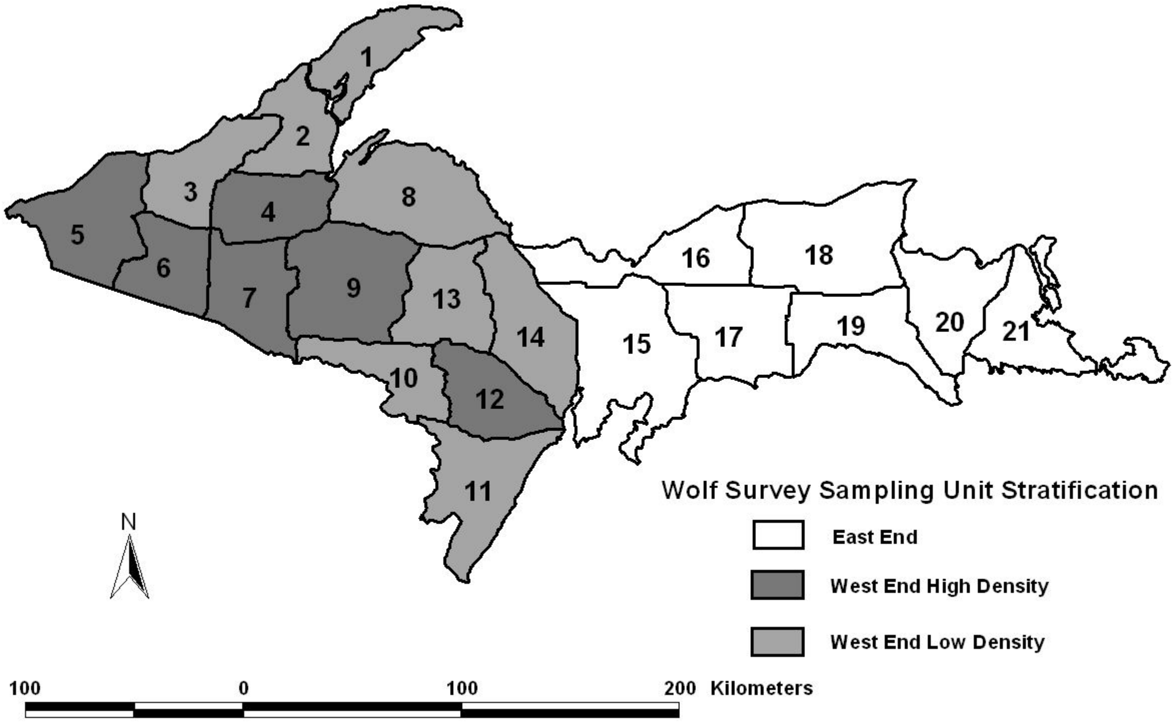

Figure 3. Wolf survey units and sampling stratification for the Upper Peninsula of Michigan.

counting of wolves whose territories overlap those boundaries. In the field, the same ground tracking and aerial observation techniques used in the past are applied to the survey units selected for sampling in a given year.

The precision of the abundance estimate can be increased over time by revisiting some sites. The correlation between counts on the same units one year apart is approximately 0.8. It is possible to take advantage of this correlation by using a panel design (Schreuder et al. 1993). A panel consists of a set of sampling units common to at least two successive surveys. The abundance estimator based on the sampling scheme can be combined with a regression type estimator that uses the correlation between successive counts to produce a single estimate with better precision (Schreuder et al. 1993). A partial replacement panel can be used to guarantee a sufficient sample for the regression estimator (Table 1). The computation of the abundance estimator using a panel survey is complex and requires more analysis than the independent sample-based estimator, but it is generally believed to be a better approach for estimating trend and change (Urquhart and Kincaid 1999).

Lower Michigan

Wolves were eliminated from the Lower Peninsula of Michigan in the early 1900s. In October 2004, a wolf was mistakenly captured and killed by a coyote trapper in Presque Isle County. Tracks of two wolves were found in the same vicinity a few weeks later. During the next three

Table 1. Partial replacement panel design for selection of sampling units, Michigan wolf survey. Selected units for a given year are indicated by 'X'.

West End High					***West End Low***					***East End***				
**Unit**	**2007**	**2008**	**2009**	**2010**	**Unit**	**2007**	**2008**	**2009**	**2010**	**Unit**	**2007**	**2008**	**2009**	**2010**
**4**		**X**		**X**	**1**	**X**			**X**	**15**	**X**	**X**		
**5**	**X**	**X**		**X**	**2**		**X**	**X**		**16**	**X**	**X**		**X**
**6**		**X**	**X**	**X**	**3**		**X**		**X**	**17**		**X**	**X**	
**7**	**X**	**X**	**X**		**8**	**X**	**X**		**X**	**18**		**X**	**X**	
**9**	**X**		**X**		**10**		**X**	**X**		**19**	**X**			**X**
**12**	**X**		**X**	**X**	**11**	**X**	**X**			**20**			**X**	**X**
					**13**	**X**		**X**		**21**	**X**		**X**	**X**
					**14**			**X**	**X**					

winters (2005–2007), portions of the northern Lower Peninsula (NLP) were surveyed for wolf sign in late February and early March. The Michigan DNR conducted the surveys cooperatively with U.S.D.A. Wildlife Services, Little Traverse Bay Band of Odawa Indians, and Central Michigan University. These surveys had two components: 1) a prioritized area search and 2) a targeted search based on citizen reports of wolves or wolf sign. The prioritized area search was conducted using the same ground-tracking methods used in the UP. Nine survey units ranging in size from 322 to 644 km^2 (200–400 mi^2) were searched. In addition, tracks and wolf sightings reported by the public were checked. No wolves or wolf sign were found during these surveys.

In the future, the targeted search approach will be used to check for wolf presence until it is determined that wolves have re-colonized the Lower Peninsula. Once wolves have become established in the Lower Peninsula, the prioritized search area method will be implemented. To conduct the targeted search approach, a press release will be issued in late January of each year to ask citizens to report any wolves or wolf sign they encounter in the Lower Peninsula during February and early March. Credible reports will be checked in the field as soon as possible. If suspected wolf tracks are found, the number of animals will be estimated by following the tracks as far as practicable, searching for distinct trails of individual animals. Other information to collect includes date, time and location of the tracks, measurements of track size and stride length, estimated age of the track and the distance followed. The time since the last snowfall will be recorded and used to help estimate track age. Scent-marks such as urination, defecation and scratching also will be recorded to help identify territorial pairs and packs. Observations of blood in urine and raised-leg urinations provide indirect evidence of breeding. In all cases of suspected wolf tracks, attempts will be made to use digital photography to document collection of evidence.

Literature Cited

Ciucci, P., L. Boitani, F. Francisci, and G. Andreoli. 1997. Home range, activity, and movements of a wolf pack in central Italy. Journal of Zoology 243:803-819.

Fuller, T. K., L. D. Mech, and J. F. Cochrane. 2003. Wolf population dynamics. Pages 161-191 *in* L. D. Mech, and L. Boitani, editors. Wolves; behavior, ecology and conservation. The University of Chicago Press, Illinois, USA.

Huntzinger, B. A., J. A. Vucetich, T. D. Drummer, and R. O. Peterson. 2005. Wolf recovery in Michigan, 2002-05 Summary. Michigan Technological University, Houghton, Michigan.

Jedrzejewski, W., K. Schmidt, J. Theuerhauf, B. Jedrzejewska, and H. Okarma. 2001. Daily movements and territory use by radio-collared wolves, *Canis lupus*, in Bialowieza Primeval Forest in Poland. Canadian Journal of Zoology 79:1993-2004.

Mech, L. D. 1966. The wolves of Isle Royale. U. S. Department of Interior, Fauna of the National Parks of the U. S. Fauna Series number 7.

Mech, L. D., and L. Boitani. 2003. Wolf social ecology. Pages 1-34 *in* L. D. Mech, and L. Boitani, editors. Wolves; behavior, ecology and conservation. The University of Chicago Press, Illinois, USA.

Potvin, M.J., T.D. Drummer, J.A. Vucetich, D.E. Beyer, Jr., R.O.Peterson and J.H. Hammill. 2005. Monitoring and habitat analysis for wolves in Upper Michigan. Journal of Wildlife Management 69:1660-1669.

Schreuder, H.T., T.G. Gregoire and G.B. Wood. 1993. Sampling Methods for Multi-resource Forest Inventory. Wiley, NY.

Urquhart, N.S. and T.M. Kincaid. 1999. Designs for detecting trend from repeated surveys of ecological resources. Journal of Agricultural, Biological and Environmental Statistics 4:404-414.
